# Supplementary material for: Phytochemical Screening and Evaluation of Antioxidant Properties and Antimicrobial Activity against Xanthomonas axonopodis of Euphorbia tirucalli Extracts in Binh Thuan Province, Vietnam
Source: Molecules. 2021 Feb 10;26(4):941. doi: 10.3390/molecules26040941 (PMC7916649; doi:10.3390/molecules26040941)
Supplement: Supplementary file 1 [file molecules-26-00941-s001.pdf]

SUPPLEMENTARY MATERIAL

Phytochemical Screening and Evaluation of Antioxidant Properties and Antimcrobial Activity Against Xanthomonas Axonopodis of *Euphorbia Tirucalli* Extracts in Binh Thuan Province, Vietnam

Nguyen Thị My Le[1,2*], Dang Xuan Cuong[3,4], Pham Van Thinh[5], Truong Ngoc Minh[6,7], Tran Dinh Manh[8], Thuc-Huy Duong[9], Tran Thi Le Minh[2*], and Vo Thi Thu Oanh[10]

[1] Faculty of Fisheries, Ho Chi Minh University of Food Industry, 140 Le Trong Tan, Tan Phu District, Ho Chi Minh 70000, Vietnam; mylenguyenthi007@gmail.com

[2] Faculty of Biological Sciences, Nong Lam University - Ho Chi Minh City, Thu Duc 71300, Ho Chi Minh 70000, Vietnam; ttlminh@hcmuaf.edu.vn (T.T.L.M.)

[3] Nha Trang Institute of Technology Application and Research, Vietnam Academy of Science and Technology, Nha Trang 650000, Vietnam; biofoodchemtech@gmail.com

[4] Faculty of Biology, Graduate University of Science and Technology, Vietnam Academy of Science and Technology, Ha Noi 100000, Vietnam

[5] Faculty of Food Science and Technology, Ho Chi Minh university of Food Industry, 140 Le Trong Tan, Tan Phu District, Ho Chi Minh 70000, Vietnam; thinhpv@hufi.edu.vn

[6] Center for Research and Technology Transfer (CRETECH), Vietnam Academy of Science and Technology, Cau Giay district, Hanoi 100000, Vietnam; minhtn689@gmail.com

[7] Institute of Applied Mechanics and Informatics, Vietnam Academy of Science and Technology, Cau Giay district, Hanoi 100000, Vietnam.

[8] Faculty of Food Technology, Thu Dau Mot University, Thu Dau Mot City, Binh Duong 820000, Viet Nam; manhtd@tdmu.edu.vn

[9] Deparment of Chemistry, Ho Chi Minh University of Education, District 5, Ho Chi Minh 77000, Vietnam; huydt@hcmue.edu.vn

[10] Faculty of Agronomy, Nong Lam University - Ho Chi Minh City, Thu Duc 71300, Ho Chi Minh 70000, Vietnam; vtthuoanh@hcmuaf.edu.vn

* Correspondence: mylenguyenthi007@gmail.com; Tel.: +84-7030-068-107 (N.T.M.L.); ttlminh@hcmuaf.edu.vn; Tel: 0987560209

Received: date; Accepted: date; Published: date

Abstract: *Euphorbia tirucalli* is a medicine plant possessing many bioactive. The paper focused on phytochemical screening (alkaloid, flavonoid, saponin, tannin, and anthraquinone), the quantification of polyphenol and flavonoids, the activating evaluation of antioxidants and antimicrobial against *Xanthomonas axonopodis* of different extracts from *Euphorbia tirucalli* grown in Binh Thuan, Vietnam. The best activity fraction was used for purification and determining bioactive ingredients. The results showed that the phytochemical study revealed the presence of alkaloids, flavonoids, tannins, and terpenoids in ethyl acetate fraction. Saponin and anthraquinone did not present in all extracts. The content of polyphenol and flavonoid of *Euphorbia tirucalli* stem was in the range of 16.65 – 106.32 mg EqAG/g and 97.97 - 450.83 µg QE/g. Ethyl acetate fraction

showed higher amounts of polyphenol and flavonoids and antimicrobial activity against *X. axonopodis* than other fractions. Antioxidant (SC50) activity of *Euphorbia tirucalli* stem was in the range of 12.91 ± 0.70 and 528.33 ± 25.15 µg/mL. At a concentration of 5.0 mg/mL and 7.5 mg/mL, the diameter of inhibition of ethyl acetate fraction was 14.33 ± 0.76 mm and 17.87 ± 0.57 mm, respectively. MIC (minimum inhibitory concentration) was 0.156 mg/mL. Scopoletin, gallic acid, and piperic acid got MIC, corresponding to 78, 312, and 312 µg/mL, respectively. Purification of ethyl acetate fraction selected scopoletin, gallic acid, and piperic acid. Scopoletin, gallic acid, and piperic acid were found in the ethyl acetate fraction of *Euphorbia tirucalli* and exhibited the treatment of citrus bacteria canker and plant diseases.
Keywords: *X. axonopodis, E. tirucalli*, phytochemical, ethyl acetate, antibacterial activity

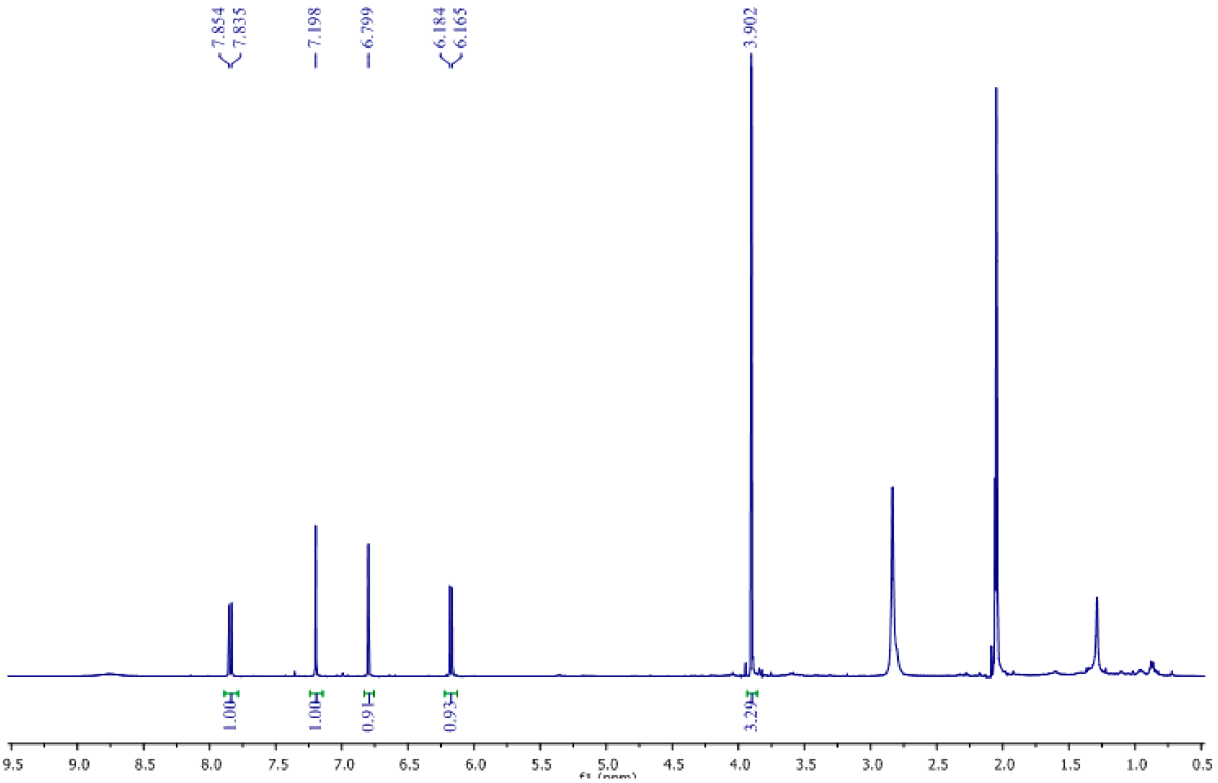

Figure S1. ^{1}H-NMR spectrum of substance 1 (scopoletin) in acetone-d_6

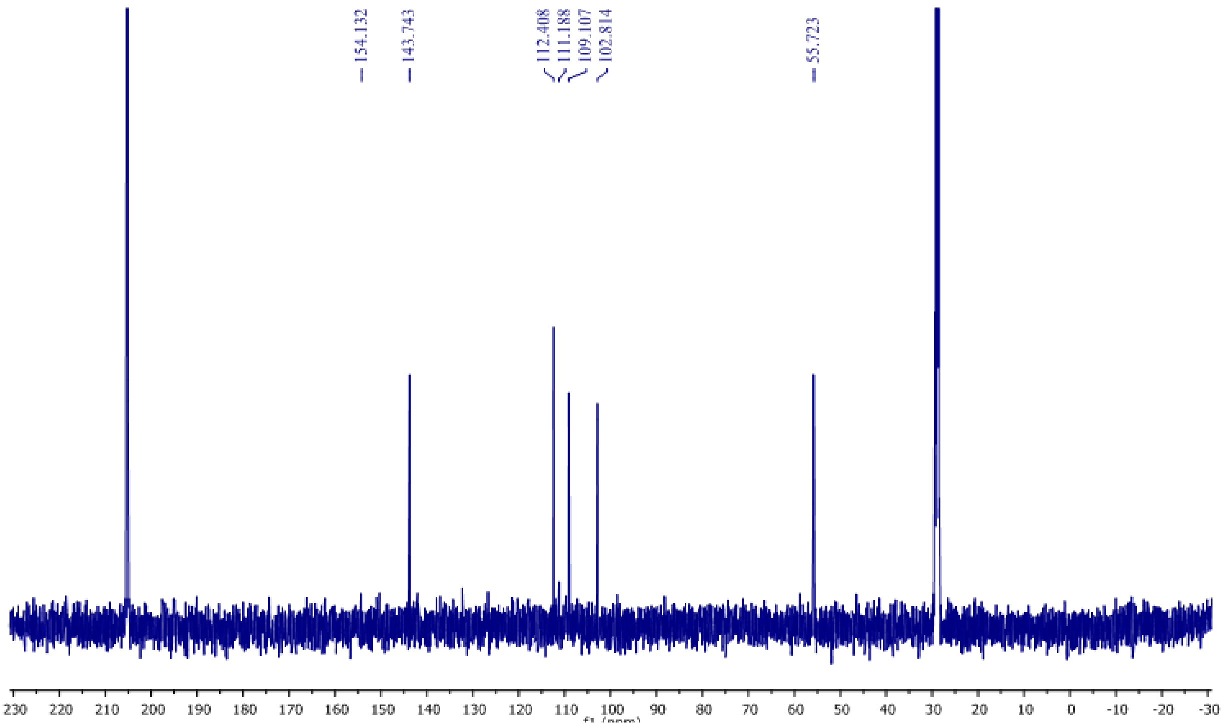

Figure S2. ^{13}C-NMR spectrum of substance 1 (scopoletin) in acetone-d_6

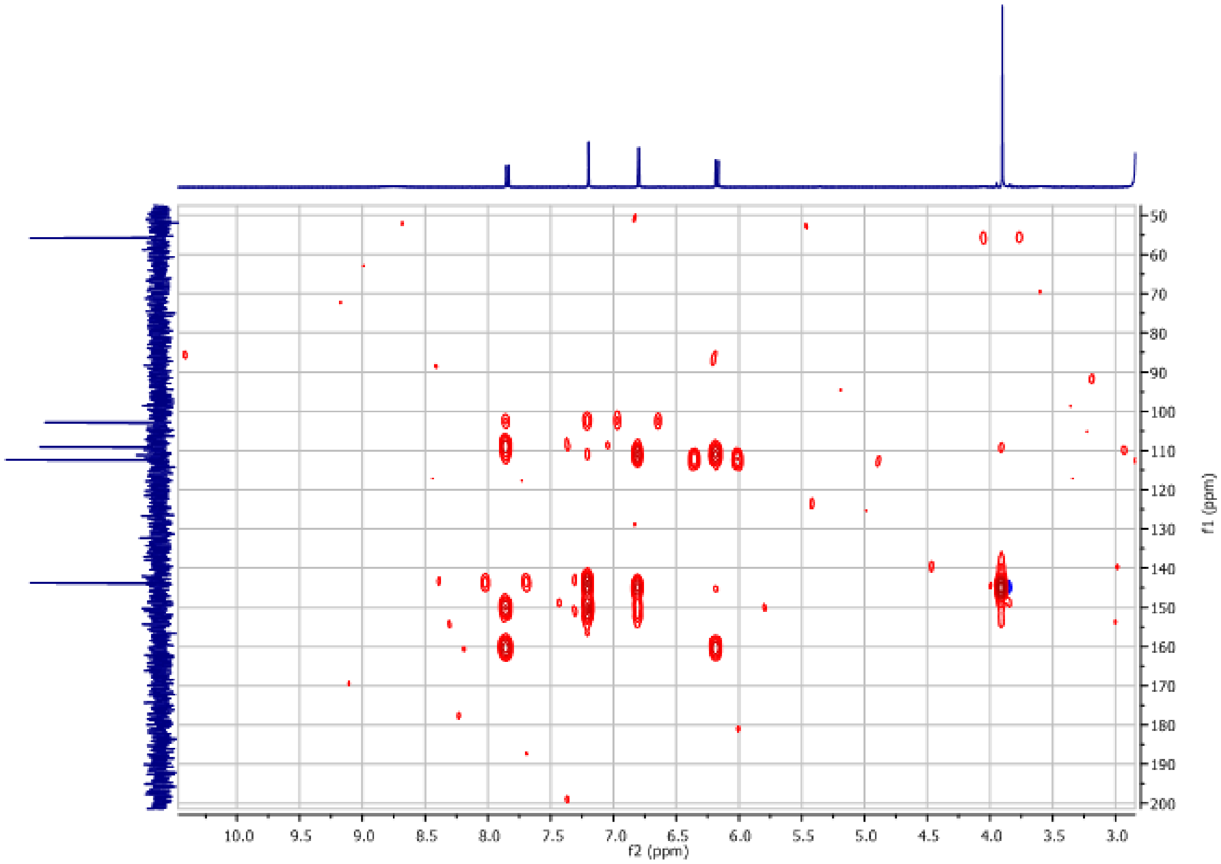

Figure S3. HMBC spectrum of substance 1 (scopoletin) in acetone-d_6

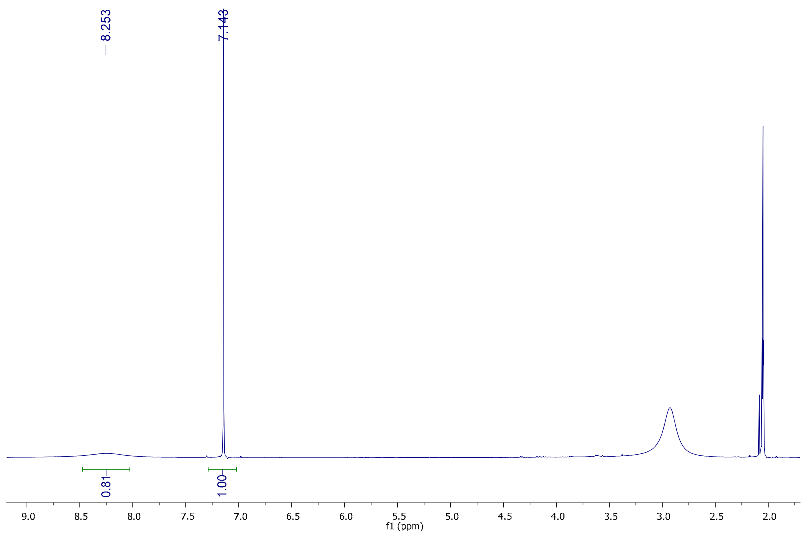

Figure S4. ^{1}H-NMR spectrum of substance 2 (gallic acid) in acetone-d_6

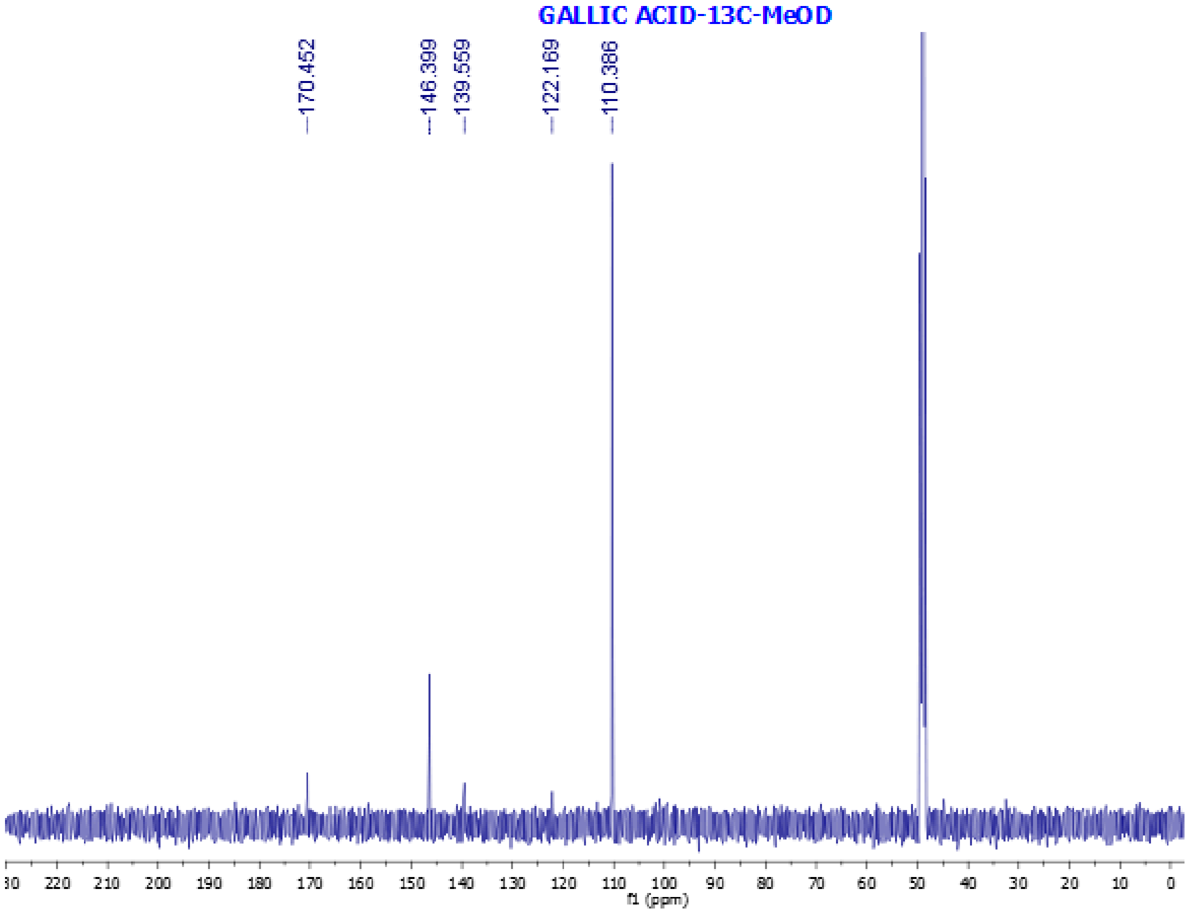

Figure S5. ^{13}C-NMR spectrum of substance 2 (gallic acid) in acetone-d_6

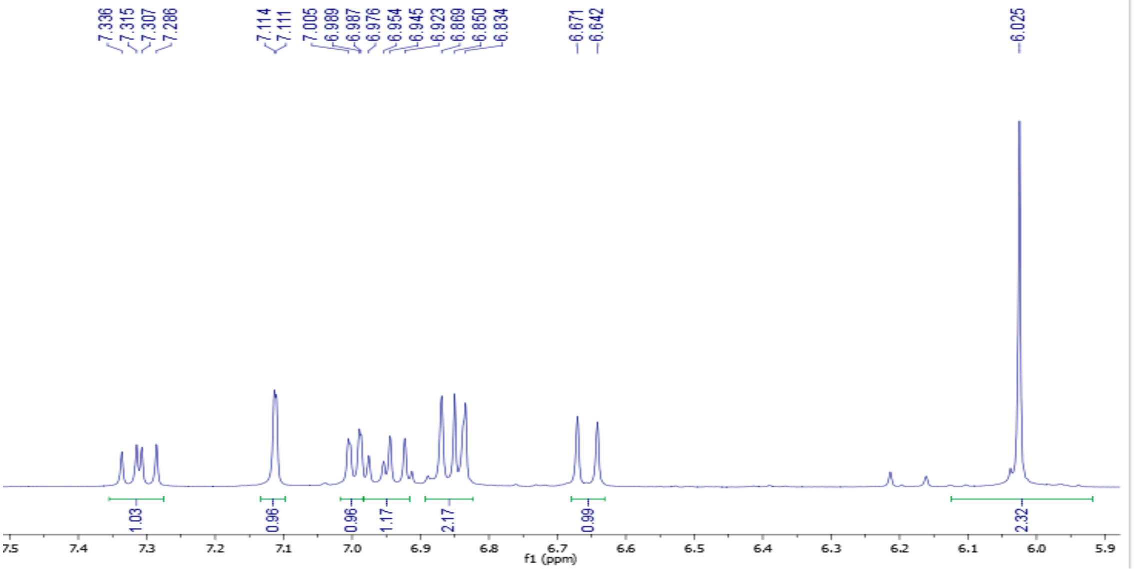

Figure S6. ^{1}H-NMR spectrum of substance 3 (piperic acid) in acetone-d_6

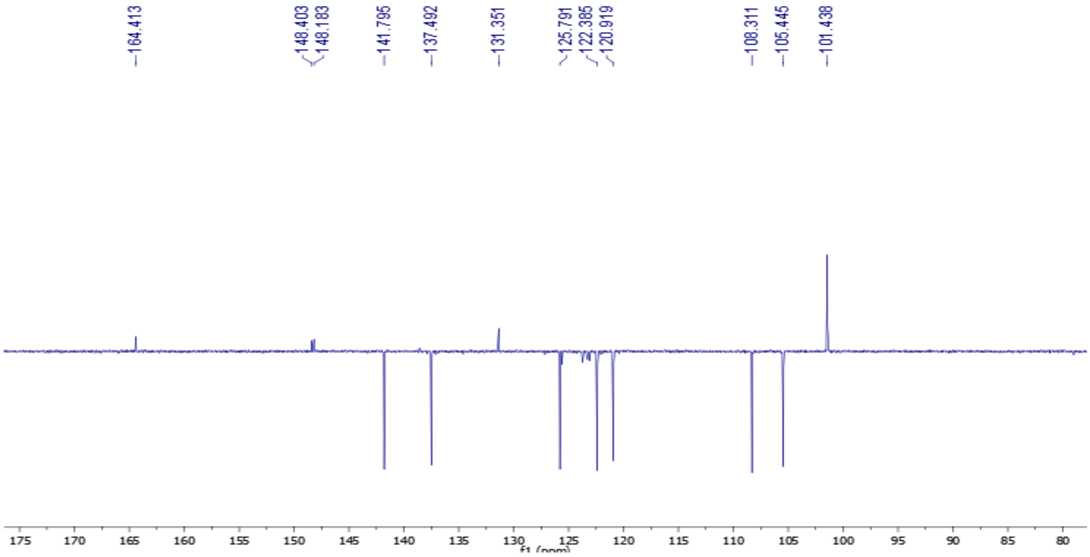

Figure S7. JMOD of substance 3 (piperic acid) in acetone-d_6

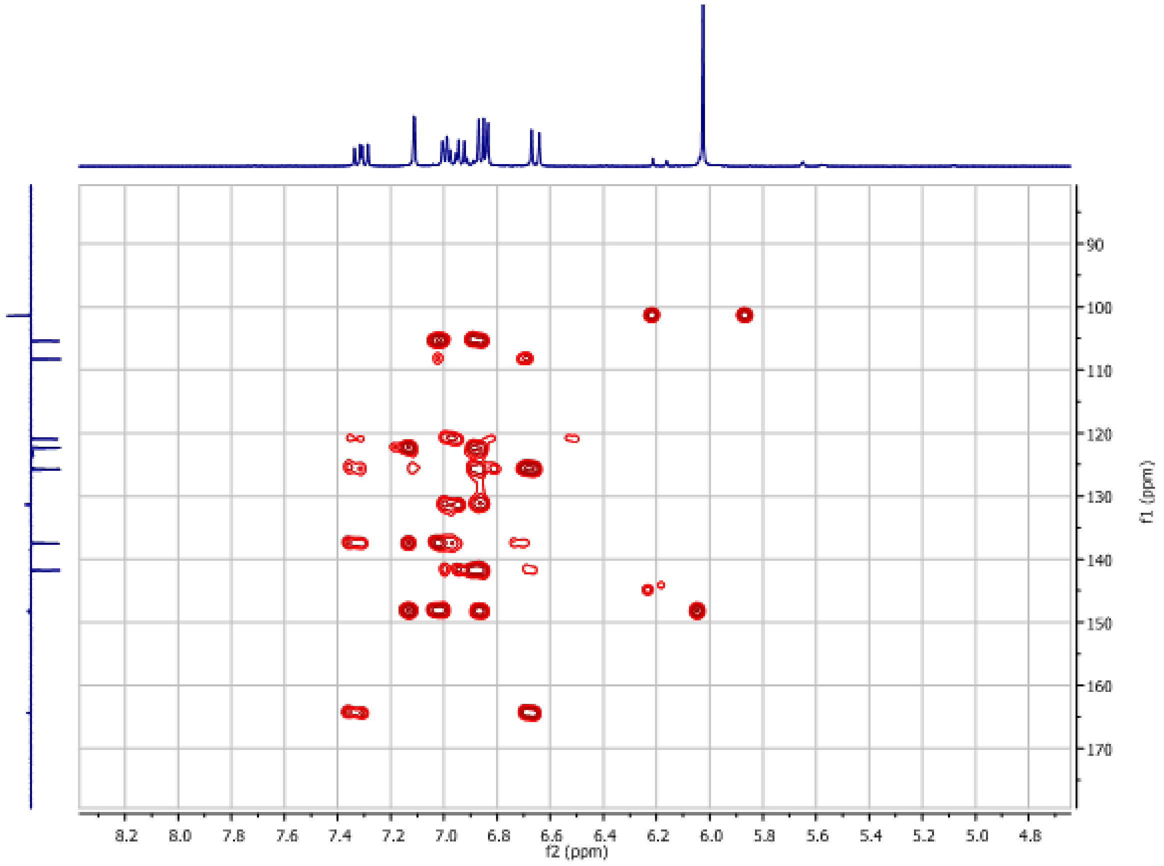

Figure S8. HMBC spectrum of substance 3 (piperic acid) in acetone-d_6
